# Characterization of *Penaeus vannamei* mitogenome focusing on genetic diversity

**DOI:** 10.1371/journal.pone.0255291

**Published:** 2021-07-30

**Authors:** Paulo Eduardo T. Soares, Márcia Danielle A. Dantas, Rita de Cássia B. Silva-Portela, Lucymara F. Agnez-Lima, Daniel Carlos F. Lanza

**Affiliations:** 1 Applied Molecular Biology Lab—LAPLIC, Department of Biochemistry, Federal University of Rio Grande do Norte, Natal, Rio Grande do Norte, Brazil; 2 Postgraduate Program in Biochemistry, Federal University of Rio Grande do Norte, Natal, RN, Brazil; 3 Laboratory of Molecular Biology and Genomics, Department of Cellular Biology and Genetics, Federal University of Rio Grande do Norte, Natal, Rio Grande do Norte, Brazil; National Cheng Kung University, TAIWAN

## Abstract

The diversity of the *Penaeus vannamei* mitochondrial genome has still been poorly characterized, there are no validated mitochondrial markers available for populational studies, and the heteroplasmy has not yet been investigated in this species. In this study, metagenomic reads extracted from the muscle of a single individual were used to assemble the mitochondrial genome (mtDNA). These data associated with mitochondrial genomes previously described allowed to evaluate the inter-individual variability and heteroplasmy. Comparison among 45 mtDNA control regions led to the detection of conserved and variable segments and the characterization of two hypervariable regions. The analysis of diversity revealed mostly low frequency polymorphisms, and heteroplasmy was found in practically all mitochondrial genes, with a high occurrence of indels. These results indicate that the design of mitochondrial markers for *P*. *vannamei* must be done with caution. The mapping of conserved and variable regions and the characterization of heteroplasmy presented here will contribute to increasing the efficiency of mitochondrial markers for population or individual studies.

## 1. Introduction

Mitochondrial genomes are short circular sequences composed of a small conserved gene repertoire and are poor in non-coding regions. These properties make the mitochondrial markers ideal for phylogenetic studies, allowing for the assessment of the population genetic structure [1]. The most common mitochondrial molecular markers are cytochrome oxidase and a non-coding region called the mitochondrial control region (CR). Inside the CR there is a highly variable sub-region called the “displacement loop” (d-loop) which is involved in mitochondrial genome replication [2, 3].

Among penaeid shrimp, the characterization of CR has only been performed for *Artemesia longinaris* and *Farfantepenaeus duorarum* [4, 5]. A study analyzing five *F*. *duorarum* populations revealed that CR presents more informative sites and haplotypes than the 16S rRNA or cytochrome oxidase subunit I gene [4]. In *Penaeus vannamei*, mitochondrial markers have been used to assess the genetic diversity in few studies. These studies presented over 40 CR haplotypes across farmed shrimp from Mexico [6, 7].

Currently, newer sequencing technologies, such as next-generation sequencing (NGS), are being applied to investigate the genetic variability in *P*. *vannamei* using shotgun sequencing approaches [8, 9], transcriptomic studies to discover SNPs associated with growth [10], genotyping for genetic selection in brood stocks [11], acquisition of high-density linkage disequilibrium maps [12], and even a combination of short reads, long reads, and BAC-end sequencing to obtain its first genomic draft [13].

Despite these advances, to date, no studies have explored the heteroplasmy in *P*. *vannamei*. In this study we explored the genetic variability of the *P*. *vannamei* mitochondrial genome and its CR at single individual (intra-individual) and inter-individual levels, characterizing their respective genetic variability, and discuss the potential impacts of the application of mitochondrial markers to study shrimp diversity.

## 2. Materials and methods

### 2.1. Data acquisition

The data used to investigate heteroplasmy were obtained from a previous study of shotgun sequencing of the caudal muscle of juvenile shrimp infected with the white spot syndrome virus—WSSV [14]. These data is available at https://www.ncbi.nlm.nih.gov/sra/PRJNA524694. To asses inter-individual diversity, sequences from four *P*. *vannamei* complete mitochondrial genomes (EF584003.1, NC_009626.1, KT596762.1, and DQ534543.1) were downloaded from GenBank. The sequence EF584003 was removed because it was curated by NCBI, receiving the new code NC_009626. In addition, 41 sequences of the *P*. *vannamei* mitochondrial control region (CR) were obtained from NCBI’s PopSet database (n = 41; https://www.ncbi.nlm.nih.gov/popset/260667508) [7].

### 2.2. Metagenomic analysis

For this analysis, raw reads were initially trimmed with Trimmomatic v0.36 [15], removing bases with a quality lower than 16 in both leading and trailing ends, and removing regions whose sliding windows of size 4 had a mean base quality inferior to 16. Trimmed data were evaluated using FASTQC(https://www.bioinformatics.babraham.ac.uk/projects/fastqc/). Duplicated reads were then removed from trimmed reads using fastx_collapser (http://hannonlab.cshl.edu/fastx_toolkit/). Taxonomical binning was performed with NCBI’s BLAST+ v2.6.0 using the BLASTn algorithm [16, 17] by aligning processed reads to GenBank’s non-redundant nucleotide database (nt). BLASTn results were visualized using Krona [18].

### 2.3. Mitochondrial genome assembly and annotation

For mitochondrial genome (mtDNA) assembly and variant calling, raw reads were trimmed using Fastpv0.19.6 [19], removing bases with a PHRED score below 20 at both ends *(--cut_front 20 --cut_tail 20*) and removing reads with size inferior to 50 bp (*--length_required 50*). Processed reads were then mapped to the reference *P*. *vannamei* mitochondrial genome (NC_009626.1) using the ‘*mem’* algorithm from Burrows-Wheeler Aligner [20]. Aligned reads were extracted with Samtools 1.9 [21] and used in SPAdes v3.14 [22] for *de novo* assembly of the mitochondrial genome using the *‘--careful’* parameter. Geneious r9 [23] was used to circularize the largest contig and then transfer annotations from the reference sequence using a 95% similarity threshold. The read coverage depth of the assembly was obtained directly from the SPAdes’ assembled scaffold sequences. Detailed mapping statistics of the assembly is presented in S1 File.

### 2.4. Alignment of the mitochondrial genomes and control region sequences

The four complete mitochondrial genomes (mitogenomes) were rotated to a common starting point using the software MARS [24]. The rotated mitogenomes were then aligned with the other 41 control region sequences using MAFFT online v7.423 [25] (https://mafft.cbrc.jp/alignment/software/), using the option ‘*adjust direction according to the first sequence’*. The mtDNA assembled in this study or its control region were used as first sequence in both MAFFT alignments. The control regions from the complete mitogenomes were then extracted from the alignment using Geneious and re-aligned with the other 41 CRs via MAFFT. An alignment with only the four rotated mitogenomes was also performed using the same MAFFT parameters.

### 2.5. Characterization of conserved and variable segments in the *P*. *vannamei* mitochondrial control region

The alignment of the 45 CRs in MAFFT was used to detect conserved regions with DnaSP v6.12 [26] adopting the “*Conserved DNA regions…*” analysis. The following *user-defined* parameters were provided: “*Minimum window length*” set to 20 and “*Conservation threshold*” set to 100. The regions found were labeled as conserved segments (CS) and the remaining regions were considered variable segments (VS). We considered CSs with a p-value less than 0.05.

### 2.6. Metrics of genomic variability

To measure genomic variability, we used three parameters: (1) the number of variable sites per gene, distinguishing the genes with more polymorphic sites. (2) a variability index (VI) to express variability as a proportion of polymorphic sites per gene length (VI = n° of polymorphic sites/gene length); since, by chance, one can expect that longer genes will have more polymorphic sites. (3) the distribution of the number of polymorphic sites over frequency intervals,to distinguish the frequency of each variant in each respective gene.

In the case of both inter-individual analyses, which use multiple sequence alignments, the table generated by Geneious’ SNP/polymorphism detection includes reference alleles as variants, distorting the variant count in a few situations. To prevent this, the following criteria were adopted: (1) variants with frequencies above 50% were assumed as reference alleles and, therefore, were not considered as variants for the given site; (2) in cases of bi-allelic sites, where both variants have a 50% frequency, the site would be considered as having a single variant since one should be the reference allele; (3) in cases of tri- and tetra-allelic sites, the most frequent allele was also considered the reference and the others, variants. To prevent that multiple variants assume the same frequency interval, we used an weighted distribution of the variant alleles per site. (4) classification of polymorphic sites in transversion-containing, transition-containing, and/or indel-containing sites to assert the types of variability found in genes.

### 2.7. Measurement of inter-individual variation

Inter-individual variability analysis was determined based on the alignment of the four mitogenomes and 41 CRs. In both cases, the Geneious’ “*Find Variations/SNPs* …” function was used using “*minimum variant frequency*” set to 0 and unchecking the “*Maximum Variant P-value*” and “*Minimum strand-bias P-value*” filters. P-values were obtained using “*Calculate Variant P-values*” option assuming a PHRED quality of 20 and using a 30% penalty because the reads were acquired from an Ion Torrent.

### 2.8. Measurement of heteroplasmy

Trimmed reads were mapped back to the assembled mitogenome (mtDNA-BR) using BWA’s “mem” algorithm. Variants were first called using Samtools v1.9 “mpileup” command using the “-B” parameter and providing the assembled mtDNA as reference to the “-f” parameter. The output was then piped to VarScan 2.3.9 (KOBOLDT *et al*., 2012), where variants were called using the parameter “mpileup2cns” with the following parameters: --*min-reads2* 4 --*min-freq-for-hom* 0.5 --*min-var-freq* 0 --*output-vcf*. The resulting VCF file was imported and visualized in Geneious, obtaining the read coverage, and variants from the annotation track “not called for any genotype” were not taken into consideration in our analysis.

### 2.9. Phylogenetic analyses

Phylogenetic analyses were performed from the alignments obtained using MAFFT. The UPGMA phylogenies were obtained using MEGA version X [27] and Bootstrap N = 1,000. The parameters for the 4 mtDNAs alignment were: Tamura-Nei model; “Substitutions to include” = d: Transitions + Transversions; “Rates among sites” = uniform; Gaps/Missing Data treatment = Pairwise deletion. The parameters for the 45 CRs alignment were: Tamura 3-parameter model; “Rate among sites” = gamma distributed (G); “Gamma parameter” = 0.18; “Pattern among lineages” = same (homogeneous);

## 3. Results

### 3.1. Assembly of *P*. *vannamei* mitochondrial genome and cluster analyses

**Fig 1:**
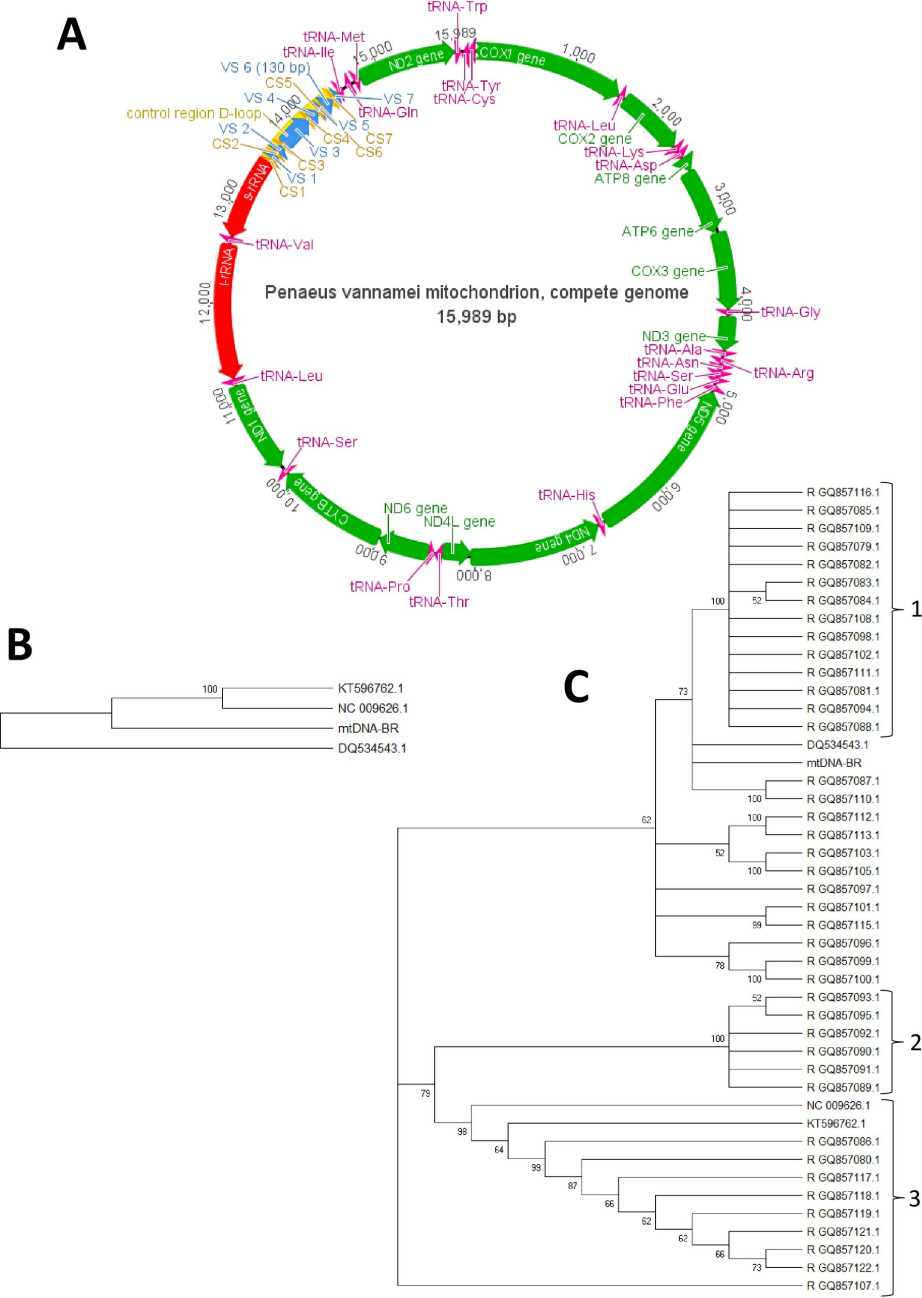
*Penaeus vannamei* mitochondrial genome. (A) The complete *Penaeus vannamei* mitochondrial genome assembled from metagenomic reads in this study (mtDNA-BR). The 7 conserved segments (yellow) and 7 variable segments (blue) were enumerated from the 5’ to 3’ of the mitochondrial sequence. The tRNA (purple) and protein coding genes (green) were also presented. (B) UPGMA phylogeny comparing full mtDNAs and (C) control regions (Bootstrap N = 1,000). The numbers 1, 2 and 3 indicate the occurrence of paraphyletic and monophyletic groups. The “R” in the sequence accessions indicate that these sequences were inverted in MAFFT to match the first sequence (see methodology for details).

The mapping of reads to the reference mitogenome NC_009626.1 yielded 8.362 mapped reads with a mean coverage of 128.4 (standard deviation = 51.3). *De novo* assembly of the reads resulted in a large contig (15.989 bp, 43.9 × mean coverage according to SPAdes) and three short contigs (< 260 bp) with mean coverages of 34.34, 56.13, and 1.5x. The large contig obtained comprised all annotations in accordance with the reference *P*. *vannamei* mitochondrial sequence (Fig 1A). The mtDNA-BR had similar characteristics to previously described mitochondrial genomes (Table 1).

**Table 1:** Genomic information of the available complete mitochondrial genomes.

Genome /Accession ID	Source	Length (bp)	GC Content (%)	CDS	rRNAs	tRNAs	CR Length (bp)
mtDNA-BR	*This study*	15989	32.3	13	2	22	995
EF584003*	*Unpublished*	15990	32.3	13	2	22	995
KT596762	[28]	15989	32.3	13	2	22	985
DQ534543	[29]	15989	32.2	13	2	22	998

* The sequence EF584003 was curated by NCBI, receiving the new code NC_009626.

UPGMA clustering of the whole mitochondrial genomes (Fig 1B) and of the 45 CRs (Fig 1C) corroborates that the genomes NC_009626 and KT596762 are the most similar. Considering the information regarding CR, three groups can be considered. Among these, the group 3 had greater sequence diversity. The mtDNA-BR sequence was grouped close to group 1 with DQ534543 (Fig 1C). In some subgroups within groups 1 and 2 the resolution of the analysis was insufficient to discriminate all individuals.

### 3.2. Inter-individual variability

The alignment of the 45 CRs revealed 7 CS comprising about 20% of the CR (196 bp) and 7 VS containing 182 polymorphic sites (ps) with an average distance of 5.2 bp (std. dev. = 5.8) (Table 2). The length of the conserved regions varied greatly, ranging from 11 bp (VS5) to 354 bp (VS3). The regions with the most polymorphic sites were VS3 (95 ps) and VS6 (26 ps), while VS1 (7 ps) and VS5 (3 ps) had the least. Considering the proportion of ps per length, VS3 and VS5 were the most variable regions (VI = 27%), while VS1 was the least variable (VI = 13%) (Table 2).

**Table 2:** Characterization of variable and conserved segments according to the 45 mitochondrial CRs.

Segment	Length (bp)	Start position*	End position*	GC Content (%)	N° of Conserved sites	N° of Polymorphic sites	Variability Index (VI)
*Variable Segments*							
VS1	55	15036	15089	8.3	48	7	12.7%
VS2	89	15117	15204	22.9	70	19	21.3%
VS3	354	15228	15579	9.7	259	95	26.8%
VS4	110	15600	15708	13	92	18	16.4%
VS5	11	15736	15746	25	8	3	27.3%
VS6	130	15782	15910	10.6	104	26	20.0%
VS7	56	15935	15990	14.3	42	14	25.0%
*Conserved Segments*							
CS1	40	14996	15035	12.5	40	-	-
CS2	27	15090	15116	14.8	27	-	-
CS3	23	15025	15227	17.4	23	-	-
CS4	20	15580	15599	25	20	-	-
CS5	27	15709	15735	29.6	27	-	-
CS6	35	15747	15781	22.9	35	-	-
CS7	24	15911	15934	16.7	24	-	-

The sequences of each variable and conserved segment can be found in S1 File.

*Position relative to the reference sequence NC_009626.1.

**GC content ignoring gaps and indel sites.

The analysis of the distribution of the variable sites per frequency interval revealed that most variants occurred in the lowest frequency interval (Table 3). The most prevalent polymorphisms were transition-containing sites (143 ps; 78.3%), followed by transversion-containing sites (16 ps; 8.8%), sites containing alleles for both transition and transversions (11 ps; 6%), indel-containing sites (9 ps; 4.9%), and lastly, site containing alleles for indels and transitions (3 ps; 1.6%) (S1 Table).

**Table 3:** Inter-individual variability in the 45 mitochondrial CRs.

Segment	(0, 10]	(10, 20]	(20, 30]	(30, 40]	(40, 50]	Total variants found	Length (bp)	Variability Index
Variable Segment 1	7.0	0.0	0.0	0.0	0.0	7	55	13%
Variable Segment 2	11.0	4.0	1.0	0.0	3.0	19	89	21%
Variable Segment 3	54.8	14.8	8.3	9.5	7.5	95	354	27%
Variable Segment 4	10.0	1.5	1.0	1.5	4.0	18	110	16%
Variable Segment 5	2.0	0.0	1.0	0.0	0.0	3	11	27%
Variable Segment 6	14.5	5.5	2.0	1.0	3.0	26	130	20%
Variable Segment 7	11.0	2.0	0.0	1.0	0.0	14	56	25%
**Total**	110.3	27.8	13.3	13	17.5	**182**	805	-
**Relative frequency**	61%	15%	7%	7%	10%	-	-	-

The values in the table were weighted according to the number of alleles found per polymorphic site and then distributed in the respective interval bin. weighted distribution is based on the number of alleles found per polymorphic sites at frequency interval (variant alleles per site per frequency interval).

Analysis of the four complete mitochondrial genomes revealed 212 ps, with an average distance of 75.4 bp (std. dev. = 148.7) (Table 4). Considering the VIs, the top 10 VS or genes included VIs ranging from 9.94 to 1,79% including all VS, and with *COX1* as the most variable gene, followed by several tRNA associated genes (Table 4). The distribution of the polymorphic sites in the frequency intervals 25% and 50% (fi_25_ and fi_50_, respectively) revealed that among the 27 genes evaluated, more variants were found within fi_25_ (168 ps) than within fi_50_ (44 ps), especially in the *COX1* gene that concentrated most of the fi_25_ variants (92 ps) (Table 4). The CR alone showed more fi_50_ variants (30 ps) than all the other genes combined (14 ps), with more than half of the variants located in VS3 (17 ps). Since all polymorphic sites were bi-allelic, they were distributed in a single category per site. Transition-containing sites were the most prevalent, corresponding to 79.7% of polymorphic sites (n = 169), followed by transversion-containing sites 17.9% (n = 38), and indel-containing sites 2.3% (n = 5) (S2 Table).

**Table 4:** Inter individual variability among the four full mitochondrial genomes.

Gene	Gene Group	25% frequency	50% frequency	Total variants found	ORF Length (bp)	VI*
VS 3	CR	18	17	35	352	9.94%
VS 5	CR	-	1	1	11	9.09%
VS 2	CR	6	1	7	87	8.05%
VS 4	CR	-	7	7	109	6.42%
COX1 gene	COX	92	-	92	1534	6.00%
VS 6	CR	4	3	7	129	5.43%
tRNA-Tyr	tRNA	3	-	3	66	4.55%
VS 1	CR	2	-	2	55	3.64%
tRNA-Glu	tRNA	-	2	2	70	2.86%
VS 7	CR	-	1	1	56	1.79%
tRNA-Ile	tRNA	1	-	1	67	1.49%
tRNA-Phe	tRNA	1	-	1	67	1.49%
tRNA-Trp	tRNA	1	-	1	69	1.45%
tRNA-Val	tRNA	1	-	1	72	1.39%
COX2 gene	COX	4	3	7	688	1.02%
ND1 gene	ND	5	2	7	938	0.75%
ND2 gene	ND	7	-	7	1000	0.70%
ND5 gene	ND	6	3	9	1723	0.52%
CYTB gene	COX	4	-	4	1136	0.35%
s-rRNA	rRNA	3	-	3	856	0.35%
ND4L gene	ND	-	1	1	300	0.33%
ND4 gene	ND	3	1	4	1341	0.30%
ATP6 gene	ATP	2	-	2	675	0.30%
l-rRNA	rRNA	2	2	4	1370	0.29%
ND3 gene	ND	1	-	1	352	0.28%
ND6 gene	ND	1	-	1	516	0.19%
COX3 gene	COX	1	-	1	790	0.13%
**Total**	27	168	44	212	14429	-
**Relative Frequency**	-	-	79.2%	20.8%	-	-

= “-” No occurrences were found or, in the case of total VI, it was a non-applicable value.

### 3.3. Heteroplasmy

Mapping the 8,496 processed reads back to the assembled mitochondrial genome revealed 186 polymorphic sites, showing an average distance of 85.9 bp (std. dev. = 99.4) (Table 5). Among these, 184 polymorphic sites correspond to indels (112 insertions and 72 deletions) and 2 were substitutions (two transitions). Detailed information is presented in S3 Table. The intervals between the polymorphic sites reached up to 548 bp, revealing large invariable regions. Among the insertions, single insertions of thymine (n = 66) and adenine (n = 44) were the majority. Among the indels, most were single-base deletions of thymine (n = 36) and adenine (n = 20). Indels involving cytosine (n = 9) and guanine (n = 6) occurred mainly in cases of deletions, representing 21% of the 72 deletions found. The largest indels found were 2 bases long (n = 3): AT and CT (insertions) and AA (deletion).

**Table 5:** Heteroplasmic variation observed in the assembled mitogenome. The frequency intervals show the distribution of the variants found according to their frequency.

Gene	Gene group	Frequency intervals of 10%						Total variants found	ORF Length	VI
		(0, 10]	(10, 20]	(20, 30]	(30, 40]	(40, 50]	(50, 60]			
ND3 gene	ND	5	7	1	-	-	-	13	352	3,69%
tRNA-Ala	tRNA	2	-	-	-	-	-	2	65	3,08%
tRNA-Arg	tRNA	2	-	-	-	-	-	2	65	3,08%
tRNA-Lys	tRNA	1	1	-	-	-	-	2	69	2,90%
CS6	CR	-	-	-	-	1	-	1	35	2,86%
ND1 gene	ND	11	9	1	1	1	-	23	938	2,45%
ATP8 gene	ATP	1	1	1	-	-	-	3	159	1,89%
VS 7	CR	-	-	1	-	-	-	1	56	1,79%
ND6 gene	ND	1	4	3	-	-	-	8	516	1,55%
tRNA-Gly	tRNA	-	1	-	-	-	-	1	66	1,52%
tRNA-Pro	tRNA	-	1	-	-	-	-	1	67	1,49%
tRNA-Thr	tRNA	-	-	-	1	-	-	1	68	1,47%
tRNA-Ser	tRNA	-	1	-	-	-	-	1	68,5	1,46%
ND5 gene	ND	19	5	1	-	-	-	25	1723	1,45%
tRNA-Asp	tRNA	1	-	-	-	-	-	1	70	1,43%
tRNA-Glu	tRNA	1	-	-	-	-	-	1	70	1,43%
CYTB gene	COX	12	3	1	-	-	-	16	1136	1,41%
ATP6 gene	ATP	8	1	-	-	-	-	9	675	1,33%
COX1 gene	COX	15	5	-	-	-	-	20	1534	1,30%
COX3 gene	COX	5	3	2	-	-	-	10	790	1,27%
VS 2	CR	-	-	-	1	-	-	1	87	1,15%
ND2 gene	ND	6	4	1	-	-	-	11	1000	1,10%
l-rRNA	rRNA	5	4	2	-	-	1	12	1370	0,88%
ND4 gene	ND	5	4	1	-	-	-	10	1341	0,75%
s-rRNA	rRNA	3	2	-	-	-	-	5	856	0,58%
COX2 gene	COX	1	2	1	-	-	-	4	688	0,58%
ND4L gene	ND	-	-	1	-	-	-	1	300	0,33%
VS 3	CR	-	-	-	1	-	-	1	352	0,28%
**Total**	**28**	**104**	**58**	**17**	**4**	**2**	**1**	**186**	**14517**	**-**

“-” = no occurrences were found or, in the case of total VI, it was a non-applicable value.

The CR showed only four ps at VS2, VS3, VS7, and CS6, with the CS6 region being the most polymorphic. The top 10 VIs considering the whole mitochondrial genome ranged from 1.52% to 3.69%, with the ND3 gene as the most variable, followed by tRNA genes (Arg, Ala, Lys), two CR regions (CS6 and VS7), ND1, ND6, and ATP8 genes (Table 5).

CS6 had the second highest frequency variant (CA→C; f = 46.4%; p-value = 1.8e-5), and the other variants occurred in VS2 (CT→C; f = 32.3%; p-value = 1.9e-4), VS3 (CT→C; f = 32%; p-value = 1.9e-3), and VS7 (C→CA; f = 21.2%; p-value = 5.4e-3). The only non-CR variants with frequencies above 30% were in three genes: l-rRNA gene (TAA→T; f = 54.6%; p-value = 3.9e-56), one of the two double-base deletions found in the *ND1* gene (G→GT; f = 44.14%; p-value = 1e-18), and tRNA-Thr gene (GT→G; f = 37.5%; p-value = 4.7e-8).

In general, protein-coding genes differed from genes that do not encode proteins because their variants (almost all indels) showed a pattern of occupying a frequency interval before occupying the next one (Table 5). For example, the lowest frequency observed in protein-coding genes were between 0 and 10% while in non-coding genes the variants could directly start at a higher frequency interval (ex.: CS6, VS2, tRNA-Gly, etc.) or would start at low frequency intervals but presenting “frequency gaps” (i.e.: l-rRNA has no variants at ~30–50% frequencies). The only exception to this was the *ND4L* gene having a single insertion with a 23.7% frequency.

### 3.4. Comparison of inter- and intra-individual variability in the mitogenome

**Fig 2:**
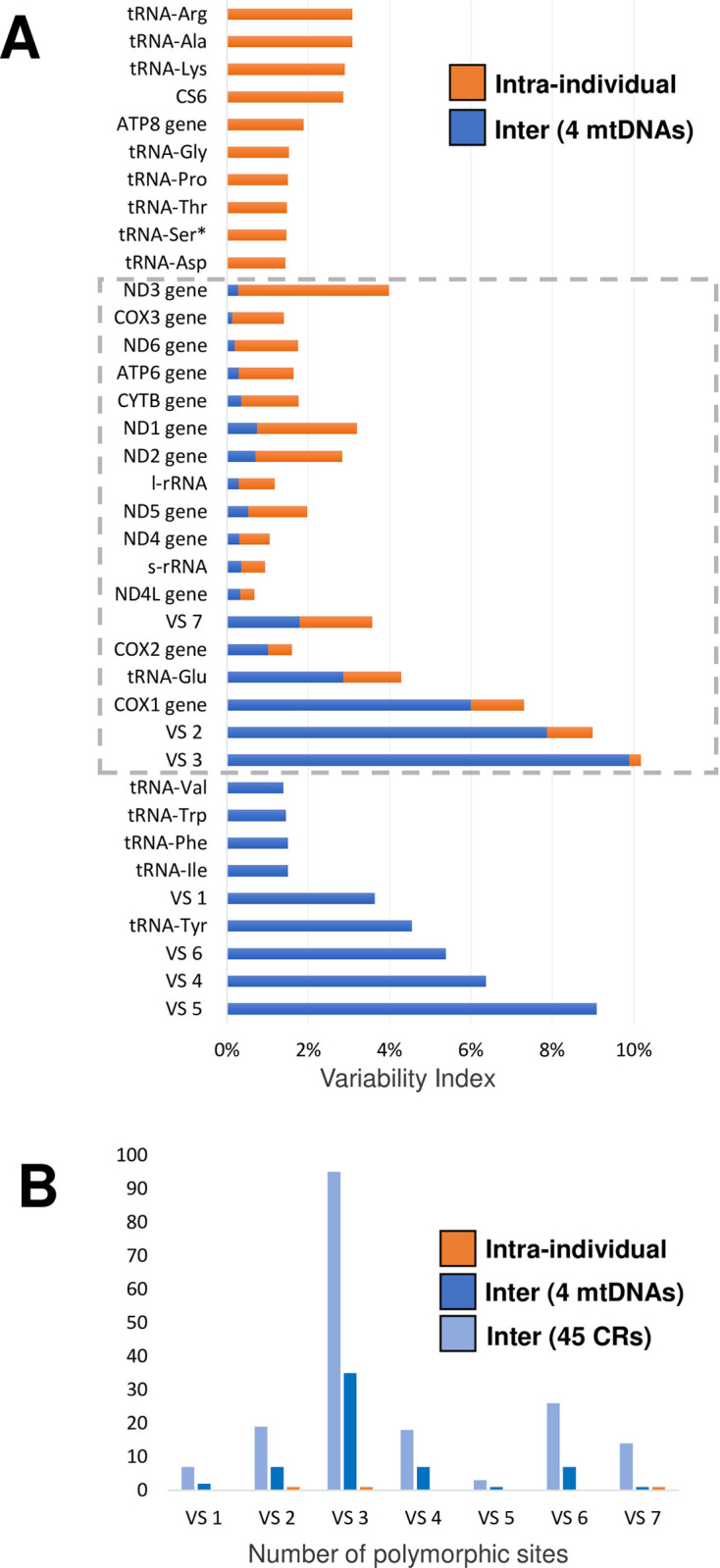
Comparative analysis of intra and inter-individual diversity. (A) Comparison of variability index (VI) considering the complete mtDNA sequences (orange) or heteroplasmy (blue). The VI represents the proportion of polymorphic sites found ie each gene. The regions that presents inter and intra individual variability are highlighted by the dotted lines. (B) The bimodal pattern generated by the number of polymorphic sites found in VS showing the peaks in VS3 and VS6.

Among the 37 genes/regions analyzed, 9 showed only inter-individual variation, 10 showed only intra-individual variation, and 18 showed variation in both individuals and within the same individual (Fig 2A). In general, the variable regions differ more inter-individually, and the genes for tRNAs vary more intra-individually. Considering the sum of the VIs of the two analyses, the VS3 region had the largest and the *NDL4* gene had the least number of variations among all the analyzed genes/regions. Among the 13 genes that encode proteins, only *COX1* and *COX2* showed inter-individual variation higher than intra-individual variation. The VS7 region and *ND4L* gene showed the same VI inter- and intra-individually.

Considering the inter-individual analysis, VS3 and VS6 were the segments with the largest number of variations. A bimodal pattern around these two VS was observed, indicating two distinct hyper variable regions (Tables 2 and 3 and Fig 2B).

## 4. Discussion

This work was motivated by the great deficiency that still exists in the characterization of mitochondrial markers for *P*. *vannamei*. It was possible to explore heteroplasmic (intra-individual) and species-wide (inter-individual) variabilities, using full genomes and partial sequences already available, and a metagenome from a muscle sample.

As expected, the phylogenetic analysis using the complete genomes showed well-defined groups (Fig 1B). However, a greater number of full genomes will be needed to confirm their efficiency in population studies and individual discrimination.

CR phylogeny presented relative consistency with the full genome analysis, but it did not bring necessary information to individual identification (Fig 1C). The 41 CR sequences used in this study were from haplotypes found in broodstocks of five hatcheries from Mexico and were found to descend from a recent common ancestor [6, 7]. This fact and the low difference among the sequences in the polyphyletic branches can explain the polyphyletic groups within the haplotype sequences. However, DQ534543.1 [29] and mtDNA-BR also clustered in this same polyphyletic group, suggesting a recent common ancestor among them.

The presence of CS and VS indicates differential selective pressures in CR. Considering the inter-individual analysis, VS3 and VS6 were the segments with the largest number of variations. A bimodal pattern around these two VS was observed, indicating two distinct hyper variable regions (Tables 2 and 3 and Fig 2B). Similarly, regions known as “hyper variable segments” (HVS) characterized in humans were often used to haplotype individuals [30]. Our results suggest that *P*. *vannamei* CR also contains two highly informative HVS: a more variable HVS1 (ranging from VS1 to VS4) and HVS2 (from VS5 to VS7) (Fig 2B).

In the intra-individual analysis, the CR had a lower read coverage than other regions of the mitogenome (~23x), probably due to its high mutation rate, which makes detection by similarity unfeasible. This possibility is corroborated by the low variation observed in the CR (only four indel variants were found) but with high frequency (20% to 46%), which unexpectedly included the CS6. The high frequency variant from CS6 could also indicate a differential selective pressure at the muscular tissue level. In fact, the metabolic diversity in tissues can exert different pressures on their mitochondria [31]. This also raises the possibility of direct or indirect influence in the observed intra-individual variation by the concurrent WSSV infection, requiring further studies.

The amount of indels identified, especially in the intra-individual analysis, caught our attention. It was no possible to verify the impact of these indels on the ORFs, since there are stop codons even considering the sequences already annotated. In agreement, Liu et al, suggest that *P*. *vannamei* mitochondrial genomes often use ATN as start codons [28]. These data show that the identification of mitochondrial genes in *P*. *vannamei* is not complete, and it is not possible to determine precisely which mutations influence each protein.

In the intra-individual analysis, a distinct frequency pattern of indels for protein-coding and non-coding genes was observed, indicating distinct selective pressures among them (Table 5). In general, protein-coding genes had variants (mostly single-base indels) that progressively regressed with increasing frequency intervals, whereas in non-coding genes, they were up to 40% more frequent than the second most frequent indel or were simply lone super frequent indels (i.e.: CS6). This indicates that small indels need to accumulate in protein-coding genes before increasing their frequency, probably by reducing their deleterious effects. In other words, this suggests that the occurrence of high frequency indels in coding genes requires other indels, perhaps to correct their frame shifts. It is most likely that the occurrence of ’solitary’ indels is more disadvantageous for protein-coding genes than for non-coding genes (rRNA, tRNAs, and mtDNA control region). In fact, it was observed that purifying selection is stronger for indels found in genes related to elementary biochemical reactions [32]. Hence, this raises concern about which type of gene (coding or non-coding) is being used to trackindividual or populational variabilities.

Other factors affecting the observed intra-individual variation can be PCR errors, neutral mutations, or genomic paralogs that cannot be distinguished by the presented methodology [33, 34]. On the other hand, despite their eventual presence, these confounding factors did not affect the “invariable regions” observed in this study, which reached up to 548 bp. In the presence of these multiple factors, the length of these invariable regions would be shorter. This suggests that these invariable regions are *loci* under strong selective pressure. These regions could even influence the variation observed in other regions. Therefore, these results suggest the potential of using NGS to find regions under selective pressure, even without prior knowledge.

Outside the CR, genes with low-frequency variants were more abundant in both analyses (Tables 3–5 and Fig 2). Most of the genes encoding proteins analyzed showed greater intra-individual variation than inter-individual variation. This can be explained by the small number of mitochondrial sequences available, and by the fact that, generally, the diversity of these sequences is usually suppressed because only the most frequent allele could be represented during the sequence assembly. Despite this, greater inter-individual diversity was observed in the COX1 and COX2 genes. However, COX1 variants were low in frequency, indicating a high mutability but low retention of variants in this gene. The large variation in the COX1 and COX2 genes, also corroborates the fact that these genes may be related to adaptation to their cellular environment or still, to the WSSV infection, known to upregulate genes related to oxidative respiration to increase energy and, supposedly, raising the survival of infected cells to oxidative stress [35].

## 5. Conclusions

The existence of intra-individual variation indicates that the use of CR as a marker for genetic and phylogenetic identification should be adopted with caution. In this context, the mapping of conserved and variable regions presented in this study will contribute to increasing the efficiency of markers for population and/or individual studies.

The possibility of the occurrence of an indel affecting the frequency of others is also a point to be considered when applying mitochondrial markers. This behavior, associated with the fact that most of the variations found are of low frequency, indicates that most of the identified polymorphisms do not have the appropriate characteristics to be used as SNPs.

## Supporting information

S1 File Mitochondrial genome assembly. (A) Read coverage of the assembled *Penaeus vannamei* mitochondrial genome. (B) Read coverage statistics from the whole mtDNA. (C) Pairplot of 5 variables in the variant call format (VCF) generated by VarScan 2.3.9. PVAL = P-value from Fisher’s Exact Test; FREQ = Variant allele frequency; RD: Number of reads supporting reference base; AD = Number of reads supporting variant base; Coverage = the sum of AD and RD, representing the full read coverage for a given site containing variants.
(PPTX)

S2 File (RAR)

S1 Table Types and frequency of polymorphic sites found in the alignment of the 45 CRs. (XLSX)

S2 Table Types and frequency of polymorphic sites found in the alignment of the 4 complete mtDNAs (inter-individual analysis). (XLSX)

S3 Table Types and frequency of polymorphic sites found in the assembled mtDNA (intra-individual analysis). (XLSX)
