# Supplementary material for: Characterization of Penaeus vannamei mitogenome focusing on genetic diversity
Source: PLoS One. 2021 Jul 30;16(7):e0255291. doi: 10.1371/journal.pone.0255291 (PMC8323954; doi:10.1371/journal.pone.0255291)
Supplement: S1 File — (A) Read coverage of the assembled Penaeus vannamei mitochondrial genome. (B) Read coverage statistics from the whole mtDNA. (C) Pairplot of 5 variables in the variant call format (VCF) generated by VarScan 2.3.9. PVAL = P-value from Fisher’s Exact Test; FREQ = Variant allele frequency; RD: Number of reads supporting reference base; AD = Number of reads supporting variant base; Coverage = the sum of AD and RD, representing the full read coverage for a given site containing variants. (PPTX) [file pone.0255291.s001.pptx]

## Slide 1
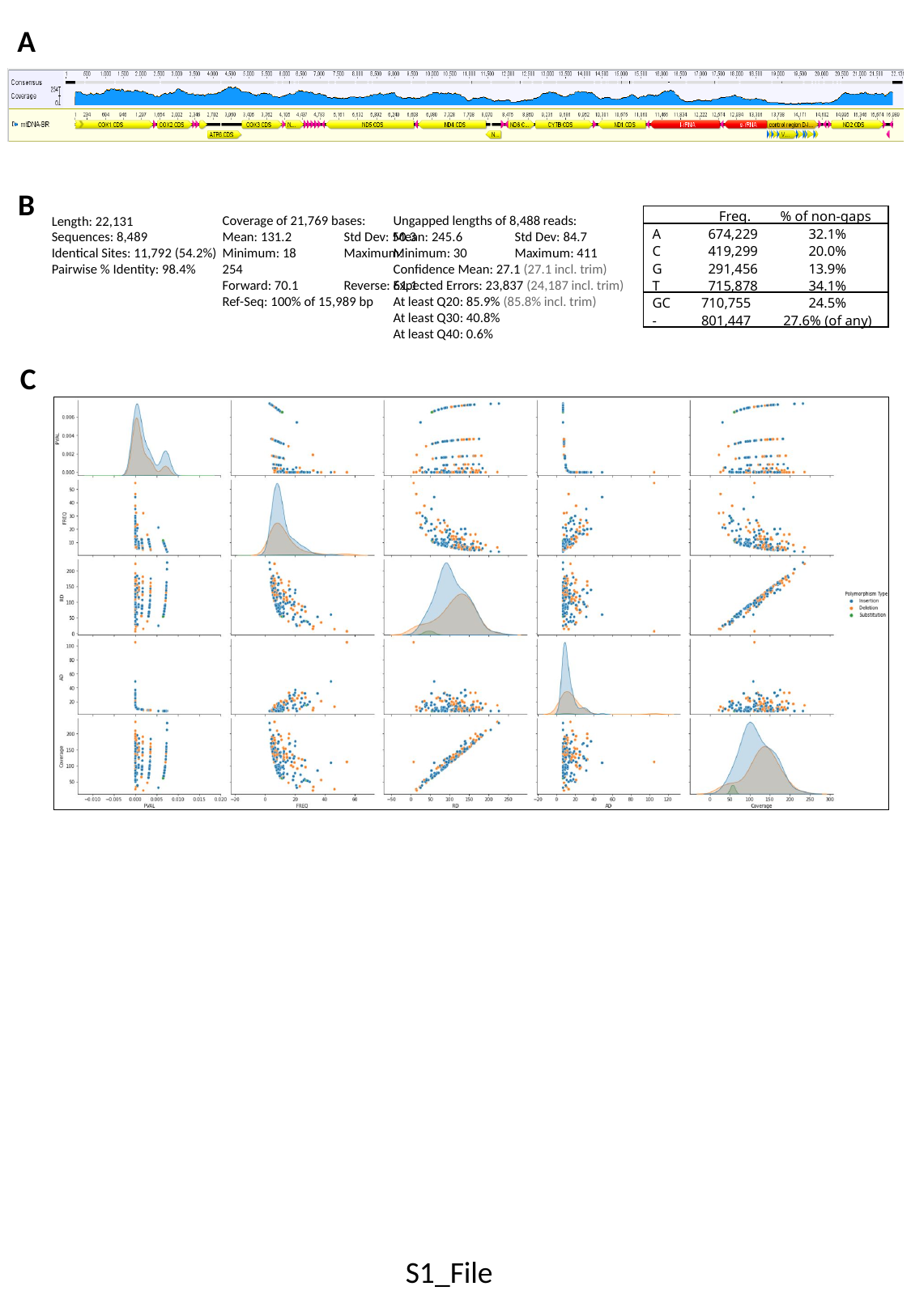

A
B
Length: 22,131
Sequences: 8,489
Identical Sites: 11,792 (54.2%)
Pairwise % Identity: 98.4%
Coverage of 21,769 bases:
Mean: 131.2	Std Dev: 50.3
Minimum: 18	Maximum: 254
Forward: 70.1	Reverse: 61.1
Ref-Seq: 100% of 15,989 bp
Ungapped lengths of 8,488 reads:
Mean: 245.6	Std Dev: 84.7
Minimum: 30	Maximum: 411
Confidence Mean: 27.1 (27.1 incl. trim)
Expected Errors: 23,837 (24,187 incl. trim)
At least Q20: 85.9% (85.8% incl. trim)
At least Q30: 40.8%
At least Q40: 0.6%
| | Freq. | % of non-gaps |
| --- | --- | --- |
| A | 674,229 | 32.1% |
| C | 419,299 | 20.0% |
| G | 291,456 | 13.9% |
| T | 715,878 | 34.1% |
| GC | 710,755 | 24.5% |
| - | 801,447 | 27.6% (of any) |
C
S1_File
